# Coagulation and fibrinolysis biomarkers associated with gastrointestinal bleeding in children with immunoglobulin A vasculitis: a retrospective cohort study

**DOI:** 10.3389/fped.2026.1886693

**Published:** 2026-10-14

**Authors:** Fang Chen, Hanjun Shen, Xuejun Shao, Jia Wei

**Affiliations:** Department of Laboratory Medicine, Children’s Hospital of Soochow University, Suzhou, Jiangsu Province, China

**Keywords:** gastrointestinal (GI) bleeding, immunoglobulin A vasculitis, plasmin-α2-plasmin inhibitor complex (PIC), serum immunoglobulin G (IgG), thrombin-antithrombin complex (TAT)

## Abstract

**Objective:**

This study aimed to explore alterations of soluble thrombomodulin (sTM), thrombin-antithrombin complex (TAT), plasmin-α2-plasmin inhibitor complex (PIC), tissue plasminogen activator-inhibitor complex (t-PAIC), and D-dimer in children with immunoglobulin A vasculitis (IgAV). We further evaluated the associations of these hemostatic biomarkers and clinical features with gastrointestinal (GI) bleeding in this cohort of patients.

**Methods:**

A retrospective cohort study was conducted at our hospital between September 2021 and April 2025, enrolling 126 consecutive children diagnosed with IgAV. Among these, 44 children presented with GI bleeding and 20 developed overt GI bleeding. Univariate and multivariate logistic regression analyses were performed to identify associated factors for GI bleeding in IgAV children. Moreover, the threshold values were assessed by receiver operating characteristic (ROC) curve analysis.

**Results:**

There were no significant differences in age, gender, sTM and t-PAIC between GI bleeding and no-GI-bleeding groups in children with IgAV. Among IgAV children, those with GI bleeding had significantly higher neutrophils, neutrophil-to-lymphocyte ratio (NLR), fibrinogen (FIB), TAT, PIC and D-dimer levels than those without GI bleeding, along with decreased serum immunoglobulin G (IgG). Children with overt bleeding had prolonged hospital stay, higher dose of methylprednisolone administration and elevated serum IgM than those with occult bleeding. Multivariate regression analysis revealed that PIC (odds ratio [OR] = 1.888, 95% confidence intervals [CI] 1.013–3.518, *P* = 0.045; area under the curve [AUC] = 0.687) was independently associated with higher odds of GI bleeding with IgAV in childhood, and serum IgG (OR = 0.843, 95%CI: 0.737–0.965, *P* = 0.013). The AUC of combined model was 0.737.

**Conclusion:**

Increased PIC levels and decreased serum IgG are closely correlated with GI bleeding in children with IgAV.

## Introduction

Immunoglobulin A vasculitis (IgAV, formerly known as Henoch-Schönlein purpura）is a systemic small vasculitis characterized by immune complexes (Immunoglobulin A1-dominant) deposited in small blood vessels (venules, arterioles or capillaries). It commonly involves the skin, GI tract, joint, kidney, with rare central nervous system involvement (1). It is the most prevalent systemic vasculitis in children aged 4–7 years, though onset can occur at any pediatric age. The global annual incidence of IgAV ranges from 3 to 27 cases per 100,000 individuals (2). Clinical features of IgAV mainly are cutaneous purpura without thrombocytopenia, arthralgias and/or arthritis, gastrointestinal symptoms and renal damage. The symptoms of IgAV in childhood with gastrointestinal involvements primarily manifest as vomiting, abdominal pain, intestinal obstruction, mesenteric lymphadenopathy. Severe complications such as GI perforation and massive life-threatening GI hemorrhage may develop without timely clinical intervention (3). Despite the clinical significance of GI bleeding, reliable biomarkers reflecting activation of the coagulation-fibrinolysis system have not been sufficiently investigated (4–6). In addition, Li et al. reported a case of a 14-year-old boy presenting with abdominal pain and melena who was diagnosed with IgAV, accompanied by superior mesenteric vein thrombosis confirmed on mesenteric CT venography (7). Therefore, our study aimed to estimate the potential associated factors for GI bleeding in pediatric IgAV by investigating a panel of hemostatic molecular markers.

We here retrospectively analyzed levels of soluble thrombomodulin (sTM), thrombin-antithrombin complex (TAT), plasmin-α2-plasmin inhibitor complex (PIC), tissue plasminogen activator-inhibitor complex (t-PAIC), fibrinogen, D-dimer and complete blood counts (CBCs), including white blood cell count (WBC), absolute neutrophil count (ANC) and neutrophil-to-lymphocyte ratio (NLR) in 44 cases of IgAV involving GI bleeding among a total cohort of 126 IgAV children.

## Methods

### Study population

A total of 126 IgAV children who had determined GI bleeding outcomes were enrolled from the Children's Hospital of Soochow University between September 2021 and April 2025. Of these patients, 85 (67.5%) were boys, with a median age of 8 years [interquartile range (IQR), 6–10 years].

IgAV was diagnosed according to the Ankara 2008 criteria and the 2019 European Single Hub and Access point for paediatric Rheumatology in Europe (SHARE) recommendations (1, 8). The mandatory diagnostic criterion was palpable purpura on skin without thrombocytopenia, combined with one or multiple of the following symptoms: (1) leucocytoclastic vasculitis with predominant IgA deposition in a skin biopsy for atypical cutaneous purpura; (2) microscopic (and/or macroscopic) haematuria (>5 red blood cells per high-power field) with or without proteinuria (urine protein>0.3 g/24 h or urine protein: urine creatinine (UP:UC) ratio>100 mg/mmol); (3) renal biopsy; (4) acute-onset joint pain with or without swelling and/or with or without limitation of movement; (5) acute-onset diffuse abdominal pain, which may be accompanied with or without vomiting, intussusception and gastrointestinal bleeding. All patients were not received corticosteroids within one week of disease onset. Patients were excluded for the following reasons: (1) Comorbidities including other vasculitides, erythema multiforme, systemic lupus erythematosus (SLE), congenital heart disease, chronic kidney disease (CKD), or malignant neoplasms; (2) Prior surgical history at enrollment; (3) Use of corticosteroids and anticoagulants within one week of disease onset; (4) Incomplete clinical records or unavailable fecal occult blood test results. The participant selection process is shown in Supplementary Figure S1.

### Definition of GI bleeding

The 126 children in the primary analytic cohort were classified into a no-GI-bleeding group (*n* = 82) and a confirmed-GI-bleeding group (*n* = 44). Furthermore, Children with confirmed GI bleeding were further categorized as having occult bleeding (*n* = 24) or overt bleeding (*n* = 20).

Occult GI bleeding was defined as a positive fecal occult blood test in the absence of clinically visible bleeding, including melena, hematochezia, and hematemesis. Overt GI bleeding was defined by clinically visible bleeding or a severe GI complication associated with bleeding, including melena, hematochezia, hematemesis with blood clots, or GI perforation. Among the 20 children with overt bleeding, 7 had melena, 7 had hematochezia, 5 had hematemesis with blood clots, and 1 had endoscopically confirmed GI perforation.

### Clinical data and laboratory methods

Clinical data were extracted from the hospital's electronic medical records, including gender, age, rash duration, length of hospital stay, methylprednisolone dosage and timing of coagulation sampling.

Clinical types of IgAV were classified as follows: Skin-limited type: presented with only cutaneous non-blanching purpura on palpation; Joint type: apart from skin type, joint pain and/or swelling; Abdominal type: manifested as abdominal pain, vomiting, hematochezia, melena, tumefaction of mesenteric lymph nodes, mucosal erythema, ecchymosis and GI perforation aside from skin type; Renal type: in addition to skin type, haematuria and/or proteinuria or renal biopsy; Mixed type: skin purpura together with involvement of at least two other organ systems.

Laboratory methods are described in Supplementary Table S1. The numbers of available observations for each laboratory parameters are reported in Supplementary Table S2.

### Statistical analysis

Statistical analyses were performed using SPSS software, version 27.0 (IBM Corp., USA). The Shapiro–Wilk test was used to assess the normality of continuous variables. Normally distributed data were presented as mean ± SD and were compared using Student's *t*-test. Non-normally distributed data were presented as median and interquartile range (IQR) and were compared using the Mann–Whitney *U*-test. Categorical variables were expressed as *n* (%) and were compared using the *χ*^2^ test or Fisher's exact test.

Univariate logistic regression was used to estimate associations between candidate laboratory variables and GI bleeding. Variables with statistical significance or clinical relevance were considered for multivariable logistic regression. Associations are reported as odds ratios (ORs) with 95% confidence intervals (CIs). Multicollinearity was evaluated using variance inflation factors (VIFs), with a VIF below 5 considered acceptable.

Exploratory receiver operating characteristic (ROC) curve analysis was performed to assess the discriminatory ability of PIC, serum IgG and a combined model for identifying GI bleeding. Discriminatory performance is reported as the area under the curve (AUC) with its 95% CI. Exploratory thresholds were selected by maximizing the Youden index, and the corresponding sensitivity and specificity were calculated. For the combined model, the threshold was based on the model-derived predicted probability. Sensitivity analyses were conducted to evaluate the potential influence of incomplete outcome ascertainment. A two-sided *P* value < 0.05 was considered statistically significant.

## Results

### Clinical characteristics according to GI bleeding status

As shown in Table 1, sex distribution, age, duration of exanthem, and timing of coagulation sampling were similar between children with and without GI bleeding.

**Table 1:** Clinical characteristics of children in the complete-case analytic cohort according to GI-bleeding status.

Clinical characteristics	No GI bleeding group (*n* = 82)	GI bleeding group (*n* = 44)	*χ*^2^/Z value	*P* value
Male, n(%)	55 (67.1%)	30 (68.2%)	0.02	0.899
Age, years	8.0 (6.0, 10.0)	8.0 (5.0, 10.8)	−0.02	0.984
Skin-limited type, n(%)	26 (31.7%)	4 (9.1%)	–	0.004
Jointl type, n(%)	27 (32.9%)	5 (11.4%)	7.03	0.008
Abdominal type, n(%)	16 (19.5%)	24 (54.5%)	16.22	<0.001
Reanl type, n(%)	9 (11.0%)	1 (2.3%)	–	0.163
Mixed type, n(%)	4 (4.9%)	4 (9.1%)	–	0.449
Duration of exanthem, days	3.0 (2.0, 6.3)	4.0 (1.0, 7.0)	−0.14	0.891
Duration of hospitalization, days	8.0 (7.0, 9.0)	10.0 (8.3, 15.8)	4.44	<0.001
Coagulation samples within 24 h admission, n(%)	49 (59.8%)	27 (61.4%)	0.03	0.860

A dash indicates that Fisher's exact test was used and no χ^2^ statistic is reported.

Boys accounted for 68.2% of the GI-bleeding group and 67.1% of the no-GI-bleeding group (*P* = 0.899), and the median age was 8.0 years in both groups (*P* = 0.984). Coagulation samples were obtained within 24 h after admission in 61.4% and 59.8% of children, respectively (*P* = 0.860).

Abdominal type was more frequent in children with GI bleeding than in those without GI bleeding (*P* < 0.001). Conversely, the proportions of children with skin-limited type (*P* = 0.004) or joint involvement phenotype (*P* = 0.008) were lower in the GI-bleeding group. The proportions with renal and mixed types were similar between groups. Children with GI bleeding had a longer hospital stay than those without GI bleeding (*P* < 0.001).

### Laboratory parameters according to GI bleeding status

Laboratory parameters in the primary analytic cohort are summarized in Table 2. Children with GI bleeding had higher WBC counts (*Z* = 1.98, *P* = 0.047, *r* = 0.18), ANC counts (*Z* = 2.85, *P* = 0.004, *r* = 0.26) and NLR (*Z* = 3.00, *P* = 0.003, *r* = 0.27) than children without GI bleeding. The distributions of ALC were similar between groups.

**Table 2:** Laboratory parameters of children in the primary analytic cohort according to GI bleeding status.

Parameters	No GI bleeding group (*n* = 82)	GI bleeding group (*n* = 44)	Z/t value	*P* value	Cohen's *d* (95%CI) or *r* value
WBC (10^9^/L)	10.1 (8.1, 12.1)	11.5 (8.6, 14.9)	1.98	0.047	0.18
ANC (10^9^/L)	6.1 (4.2, 8.3)	8.3 (5.3, 11.0)	2.85	0.004	0.26
ALC (10^9^/L)	2.1 (1.4, 2.8)	1.8 (1.2, 2.3)	−1.77	0.076	0.16
NLR	2.7 (1.9, 4.9)	4.5 (2.6, 6.6)	3.00	0.003	0.27
sTM (TU/mL)	7.9 ± 2.0	8.3 ± 2.3	−1.04	0.299	−0.195 (−0.562–0.173)
t-PAIC (ng/mL)	2.4 (2.0, 3.2)	2.6 (1.8, 3.9)	0.51	0.614	0.05
TAT (ng/mL)	4.4 (2.3, 7.7)	6.6 (4.9, 11.7)	3.03	0.002	0.27
PIC (μg/mL)	1.0 (0.7, 1.5)	1.5 (1.0, 2.4)	3.39	<0.001	0.30
D-dimer (μg/mL)	1.3 (0.4, 2.2)	2.5 (2.0, 6.8)	4.85	<0.001	0.43
FIB (g/L)	3.7 ± 0.8	4.0 ± 0.8	−2.04	0.044	−0.383 (−0.755 - −0.010)
C3 (g/L)	1.2 ± 0.3	1.2 ± 0.3	0.10	0.918	0.019 (−0.350–0.389)
C4 (g/L)	0.3 ± 0.1	0.3 ± 0.1	−0.410	0.683	−0.077 (−0.447–0.293)
Serum IgA (g/L)	2.1 (1.6, 2.8)	1.9 (1.6, 2.8)	−0.63	0.527	0.06
Serum IgG (g/L)	12.0 (10.0, 14.5)	10.4 (8.9, 11.8)	−2.63	0.009	0.24
Serum IgM (g/L)	1.1 ± 0.4	1.1 ± 0.4	1.12	0.266	0.211 (−0.160–0.581)

|d| value indicates effect estimate for *t*-test, r value indicates effect estimate for Mann–Whitney *U* test; |d| or r value = 0.1–0.3 indicates small-to-moderate effect, r value = 0.3–0.5 indicates moderate effect.

Available case numbers for each laboratory parameter are provided in Supplementary Table S2.

The GI-bleeding group also had higher FIB (t(125) = −2.04, *P* = 0.044, Cohen's *d* = −0.383, 95%CI[−0.755- −0.010]), TAT (*Z* = 3.03, *P* = 0.002, *r* = 0.27), PIC (*Z* = 3.39, *P* < 0.001, *r* = 0.30) and D-dimer (*Z* = 4.85, *P* < 0.001, *r* = 0.43). In contrast, sTM and t-PAIC distributions were similar between groups. Complement C3 and C4 levels were also comparable. Serum IgG was lower in children with GI bleeding(*Z* = −2.63, *P* = 0.009, *r* = 0.24), whereas serum IgA and IgM showed no significant difference between two groups. The numbers of available observations for individual laboratory parameters are provided in Supplementary Table S2.

### Logistic regression analysis for GI bleeding

In univariate logistic regression, higher ANC, PIC and D-dimer levels were associated with higher odds of GI bleeding, whereas higher serum IgG was associated with lower odds of GI bleeding (Table 3). Since WBC, ANC, NLR are strongly mathematically related and increased neutrophils have been reported in GI tissue of IgAV children, ANC was enrolled in multivariate logistic model (9). Furthermore, elevated TAT in GI bleeding group indicates *in-vivo* thrombin generation and activation of the coagulation system, which is tightly associated with the homeostatic balance of coagulation and fibrinolysis (10). Consequently, TAT was selected into multivariate logistic mode as well.

**Table 3:** Logistic regression analysis of laboratory parameters associated with GI bleeding in the primary analytic cohort.

Variables	Univariate regression analysis			Multivariate regression analysis			VIF value
	OR (95%CI)	SE	*P* value	OR (95%CI)	SE	*P* value	
ANC (10^9^/L)	1.143 (1.036–1.261)	0.050	0.008	1.097 (0.970–1.241)	0.063	0.140	1.389
TAT (ng/mL)	1.008 (0.987–1.031)	0.011	0.448	0.956 (0.903–1.013)	0.029	0.130	1.871
PIC (μg/mL)	1.603 (1.086–2.366)	0.199	0.017	1.888 (1.013–3.518)	0.318	0.045	2.199
D-dimer (μg/mL)	1.327 (1.141–1.542)	0.077	<0.001	1.191 (0.998–1.422)	0.090	0.053	1.617
Serum IgG (g/L)	0.887 (0.791–0.994)	0.058	0.039	0.843 (0.737–0.965)	0.069	0.013	1.043

In the multivariable model, each 1 μg/mL increase in PIC was associated with 1.89-fold higher odds of GI bleeding (OR = 1.888, 95% CI 1.013–3.518; *P* = 0.045). Each 1 g/L increase in serum IgG was associated with 15.7% lower odds of GI bleeding (OR = 0.843, 95% CI 0.737–0.965; *P* = 0.013).

The estimate for D-dimer remained in the direction of higher odds of GI bleeding, although its 95% CI included the null value (OR per 1 μg/mL increase = 1.191, 95% CI 0.998–1.422; *P* = 0.053). The adjusted estimates for ANC (OR = 1.097, 95% CI 0.970–1.241) and TAT (OR = 0.956, 95% CI 0.903–1.013) also included the null value.

All variance inflation factors were below 2.5, providing no evidence of substantial multicollinearity among the included variables.

### Exploratory ROC curve analysis for identifying GI bleeding

Exploratory ROC curve analysis was performed for PIC, serum IgG, and their combined model (Figure 1 and Table 4). PIC showed modest discriminatory ability for identifying GI bleeding, with an AUC of 0.687 (95% CI: 0.590–0.785; *P* = 0.001). At an exploratory threshold of 1.25 μg/mL, its sensitivity and specificity were 69.8% and 66.7%, respectively. Lower serum IgG showed weaker discriminatory ability, with an AUC of 0.633 (95% CI: 0.530–0.737; *P* = 0.015). At an exploratory threshold of 11.86 g/L, its sensitivity and specificity were 76.7% and 50.6%, respectively.

**Figure 1:**
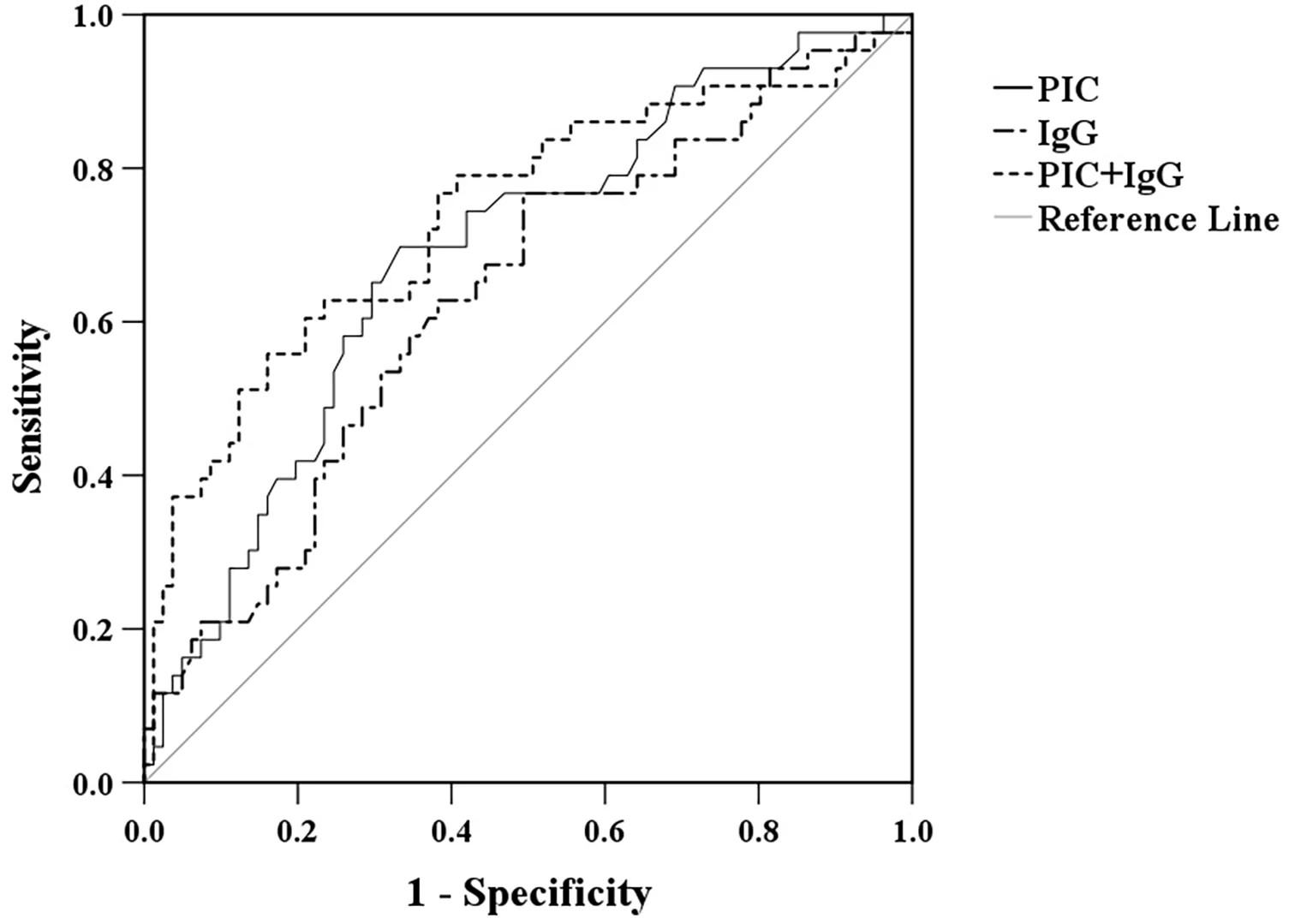
Exploratory ROC curve analysis of PIC, serum igG, and their combined model for GI bleeding.

**Table 4:** Exploratory ROC analysis of PIC, serum igG, and their combination for identifying GI bleeding.

Variables	AUC with 95%CI	SE	Youden index	Exploratory threshold value	Sensitivity	Specificity	*P* value
PIC	0.687 (0.590–0.785)	0.050	0.365	≥1.25 μg/mL	69.8%	66.7%	0.001
Serum IgG	0.633 (0.530–0.737)	0.053	0.273	≤11.86 g/L	76.7%	50.6%	0.015
PIC + IgG	0.737 (0.639–0.835)	0.050	0.398	≥0.41	55.8%	84.0%	<0.001

The combined model incorporating PIC and serum IgG yielded a numerically higher AUC of 0.737 (95% CI: 0.639–0.835; *P* < 0.001). At an exploratory predicted-probability threshold of 0.41, the model had a sensitivity of 55.8% and a specificity of 84.0%.

### Comparison between occult and overt gastrointestinal bleeding

Among the 44 children with confirmed GI bleeding, 24 had occult bleeding and 20 had overt bleeding (Table 5). Sex distribution, age, duration of exanthem, and most clinical phenotypes were similar between the two subgroups. Abdominal involvement phenotype was observed in 70.0% of children with overt bleeding and 41.7% of those with occult bleeding, corresponding to an absolute difference of 28.3 percentage points (*P* = 0.060).

**Table 5:** Comparison of clinical characteristics and laboratory parameters between children with occult and overt GI bleeding.

Variables	Occult GI bleeding group (*n* = 24)	Overt GI bleeding group (*n* = 20)	χ^2^/Z/t value	*P* value
Male, n(%)	16 (66.7%)	14 (70.0%)	0.06	0.813
Age, years	8.0 (4.3, 9.0)	9.0 (6.0, 12.0)	1.59	0.113
Skin-limited type, n(%)	4 (16.7%)	0 (0%)	–	0.114
Jointl type, n(%)	5 (20.8%)	0 (0%)	–	0.053
Abdominal type, n(%)	10 (41.7%)	14 (70.0%)	3.53	0.060
Reanl type, n(%)	1 (4.2%)	0 (0%)	–	1.000
Mixed type, n(%)	4 (16.7%)	6 (30.0%)	–	0.472
Duration of exanthem, days	2.5 (1.0, 5.0)	4.5 (2.0, 7.0)	1.42	0.154
Duration of hospitalization, days	9.0 (7.0, 9.8)	14.5 (10.8, 20.3)	4.25	<0.001
Methylprednisolone dose, mg/day	30.0 (20.0, 40.0)	40.0 (30.0, 55.0)	2.51	0.012
WBC (10^9^/L)	11.3 (8.4, 13.0)	12.0 (8.8, 16.2)	0.79	0.430
ANC (10^9^/L)	7.0 (5.3, 9.7)	9.3 (5.3, 12.9)	1.19	0.233
ALC (10^9^/L)	2.1 (1.2, 2.6)	1.5 (1.1, 2.1)	−1.44	0.149
NLR	4.0 (2.5, 5.4)	5.2 (2.7, 9.5)	1.50	0.133
sTM (TU/mL)	8.5 ± 2.0	8.1 ± 2.6	0.65	0.517
t-PAIC (ng/mL)	2.2 (1.7, 3.5)	2.9 (2.1, 4.5)	1.531	0.125
TAT (ng/mL)	7.0 (5.1, 11.9)	6.2 (3.8, 11.3)	−0.51	0.612
PIC (μg/mL)	1.8 (0.9, 2.8)	1.4 (1.0, 2.0)	−1.02	0.305
D-dimer (μg/mL)	2.5 (1.8, 6.1)	2.6 (2.1, 7.1)	0.48	0.629
FIB (g/L)	4.0 ± 0.5	4.0 ± 1.1	−0.15	0.882
C3 (g/L)	1.2 ± 0.3	1.2 ± 0.3	0.01	0.996
C4 (g/L)	0.3 ± 0.1	0.3 ± 0.1	0.91	0.369
Serum IgA (g/L)	1.9 (1.5, 2.5)	1.9 (1.6, 3.0)	0.13	0.893
Serum IgG (g/L)	10.4 (7.6, 13.7)	10.1 (9.5, 11.7)	0.22	0.827
Serum IgM (g/L)	0.9 ± 0.3	1.2 ± 0.4	−2.32	0.025

A dash indicates that Fisher's exact test was used and no χ^2^ statistic is reported.

Most laboratory parameters were not significantly different between the occult and overt bleeding groups. Children with overt bleeding had a longer length of hospital stay than those with occult bleeding (*P* < 0.001) and received a higher methylprednisolone doses (*P* = 0.012). Serum IgM was higher in the overt bleeding group than in the occult bleeding group (*P* = 0.025). These subgroup comparisons were exploratory and were not adjusted for multiple comparisons.

### Sensitivity analyses for incomplete outcome ascertainment

Sensitivity analyses evaluated the influence of alternative outcome assumptions for children with undetermined GI bleeding status (Supplementary Table S3). In the primary analysis, children with undetermined GI bleeding status were excluded. When all children with undetermined GI bleeding status were classified as having no gastrointestinal bleeding, higher ANC (OR = 1.120, 95% CI: 1.008–1.244) and lower serum IgG (OR = 0.876, 95% CI: 0.789–0.973) were associated with GI bleeding. When all children with undetermined status were classified as having GI bleeding, higher PIC (OR = 1.666, 95% CI: 1.029–2.697) and D-dimer (OR = 1.199, 95% CI: 1.027–1.399) were associated with the outcome.

The effect estimates and variables retained in the multivariable models varied across outcome-classification scenarios. Thus, incomplete outcome ascertainment may have influenced the estimated associations, and the multivariable findings should be interpreted as exploratory.

## Discussion

IgAV is the most prevalent vasculitis in childhood, with higher incidence during winter (11, 12). Compared with adult IgAV, pediatric cases are more frequently associated with GI and joint manifestations, whereas skin-limited disease and nephritis are relatively less common (13). GI symptoms, accounting for 28%–60.9% of pediatric IgAV, often manifest as vomiting, abdominal pain, abdominal distention, GI hemorrhage and ischemia (12, 13). In our study, approximately 27.2% of IgAV children developed GI bleeding. Our research demonstrated that skin-limited and joint-only phenotypes occurred less frequently among IgAV children with GI bleeding, and these patients had prolonged hospitalization.

TM is a glycoprotein that reflects endothelial cell injury, inhibits coagulation, and regulates inflammatory pathways and fibrinolysis via the activated protein C system (14, 15). Following endothelial cell damage, plasminogen activator inhibitor−1(PAI-1) is released, which counteracts tissue-type plasminogen activator (t-PA). PAI-1 binds t-PA to form the t-PA-PAI-1 complex (t-PAIC). This complex reflects sustained endothelial injury and the progression of fibrinolytic system (16). Although thrombin is hard to measure directly owing to its short half-life, thrombin forms a stable, detectable 1:1 complex with antithrombin, termed TAT, which indicates coagulation-system activation (10). In the same manner, plasmin degrades fibrin clots to yield fibrin degradation products (FDPs), among which D-dimer is a key secondary fibrinolytic marker. Once the fibrinolytic system is initiated, plasmin combines with *α*2-plasmin inhibitor (synthesized by the liver) to generate stable PIC (17). Collectively, sTM, t-PAIC, TAT and PIC are referred to as prothrombotic biomarkers or markers of a prothrombotic state. These biomarkers can predict prothrombotic status in thrombotic diseases such as disseminated intravascular coagulation (DIC) and malignancies, evaluate the prognosis of acute ischemic stroke and postoperative hemorrhage, and monitor responses to antifibrinolytic agent administration (18–23).

Compared with the no-GI-bleeding group, children with GI bleeding exhibited not only elevated WBC, ANC and NLR, but also significantly increased TAT, PIC, FIB and D-dimer, accompanied by decreased IgG levels in our research. This finding aligns with our previous report describing elevated PIC and D-dimer during the acute phase and onset of IgAV (24), implying a state of hyperfibrinolysis in IgAV children with GI bleeding. Histological data from GI biopsies of IgAV patients reported by Louie et al. also revealed lamina propria hemorrhage, fibrin deposition in the lamina propria and leukocytoclastic vasculitis (25% of specimens), without obvious endothelial necrosis (9). Combined with the findings of our current study, these observations illustrate that thromboinflammation coexists with GI bleeding in children with IgAV complicated by GI bleeding, accompanied by increased neutrophil accumulation within the vasculature or the GI tract. Notably, activation of the coagulofibrinolytic system in these children seems to be driven by neutrophils rather than endothelial injury, as evidenced by the absence of significant differences in sTM levels between the GI bleeding and no-GI-bleeding groups.

This assumption and the underlying process are also corroborated by observations from other investigations, other than that reported by Louie et al. Some studies have confirmed that IgA1 immune complexes (IgA-ICs) bind to polymorphonuclear neutrophils (PMNs) and promote neutrophils to release neutrophil extracellular traps (NETs) via Fc*α* receptor I (Fc*α* RI) (25, 26). Further investigations have demonstrated increased levels of NETs in the serum, renal, gastric and duodenal tissues of pediatric IgAV patients during the acute phase and at disease onset (27, 28).

Furthermore, Qiu et al. recently reported that neutrophils contributed to early clot resolution by releasing elastase within inflammatory microthrombi (29).

Beyond neutrophil, TAT, PIC and D-dimer elevation, we also identified decreased serum IgG concentrations in our GI bleeding group, which is consistent with findings from Yang et al. and Zhang et al. (30, 31). This indicates disturbed humoral immunity in IgAV children with GI bleeding.

Notably, serum IgG<11.86 g/L and PIC >1.85 μg/mL were identified as factors associated with GI bleeding in children with IgAV. Likewise, children with GI bleeding in the cohort reported by Yang et al. exhibited reduced IgG levels, which served as an independent predictor for GI bleeding in their research (30).

Stratified comparison between occult and overt GI bleeding revealed prolonged hospital stay, higher methylprednisolone dosage, and elevated IgM in patients with overt bleeding, reflecting a greater clinical burden and humoral immune activation in overt hemorrhage.

## Study limitations

This study has several limitations. First, our findings might be constrained by its retrospective, single-center design, as well as the relatively small sample size available for subgroup analyses, particularly GI perforation. Second, in the primary multivariate logistic regression, serum IgG was independently associated with GI bleeding. However, this association became borderline non-significant under one sensitivity-analysis scenario, indicating that this finding should be regarded as exploratory rather than definitive.

## Conclusion

We found that pediatric IgAV with GI bleeding was characterized by a hyperactive coagulation-fibrinolysis system. we identified PIC and serum IgG were independently associated with GI bleeding in this retrospective cohort. Nevertheless, all biomarker thresholds in this study were exploratory, and additional large-scale and prospective multicenter collaborative studies are necessary for external validation.

## Data Availability

The original contributions presented in the study are included in the article/Supplementary Material, further inquiries can be directed to the corresponding author/s.
