# Supplementary material for: Coagulation and fibrinolysis biomarkers associated with gastrointestinal bleeding in children with immunoglobulin A vasculitis: a retrospective cohort study
Source: Front Pediatr. 2026 Oct 14;14:1886693. doi: 10.3389/fped.2026.1886693 (PMC13713747; doi:10.3389/fped.2026.1886693)
Supplement: Supplementary file 1 [file Supplementaryfile1.docx]

**Supplementary Figure S1** Flowchart of study population enrollment

**Supplementary Table S1 Methods and performance of laboratory parameters**

| **Parameters** | **Units** | **Reference intervals** | **Analytical ranges** | **Methods** | **Reagent/instruments** |
| --- | --- | --- | --- | --- | --- |
| Complete blood counts | | | | | |
| WBC | cells/L | 0-<28 days: (15-20)*10^9/L; 28 days-<6months: (4.3-14.2)*10^9/L; 6 months-<1 year: (4.8-14.6)*10^9/L; 1-<2 years: (5.1-14.1)*10^9/L; 2-<6 years: (4.4-11.9)*10^9/L; 6-<13years: (4.3-11.3)*10^9/L; 13-18 years: (4.1-11.0)*10^9/L | (0-350) *10^9^/L | Complete blood‑count assay | Blood Cell Count Kits (Sysmex Corporation Co., Ltd., Kobe, Japan); XN‑1000 Automated Hematology Analyzer |
| ANC | cells/L | 0-<28 days: (4.65-8)*10^9/L; 28 days-<6months: (0.6-7.5)*10^9/L; 6 months-<1 year: (0.8-6.4)*10^9/L; 1-<2 years: (0.8-5.8)*10^9/L; 2-<6 years: (1.2-7.0)*10^9/L; 6-<13years: (1.6-7.8)*10^9/L; 13-18 years: (1.8-8.3)*10^9/L | - |  |  |
| ALC | cells/L | 0-<28 days: (6-12)*10^9/L; 28 days-<6months: (2.4-9.5)*10^9/L; 6 months-<1 year: (2.5-9)*10^9/L; 1-<2 years: (2.4-8.7)*10^9/L; 2-<6 years: (1.8-6.3)*10^9/L; 6-<13years: (1.5-4.6)*10^9/L; 13-18 years: (1.2-3.8)*10^9/L | - |  |  |
| Coagulation parameters | | | | | |
| sTM | TU/mL | 3.8-13.3 TU/mL | 1-200 TU/mL | sTM, t-PAIC, TAT, PIC: chemiluminescence enzyme immunoassay; D-dimer: latex-enhanced immunoturbidimetric assay; FIB: Clauss clotting assay. | sTM/t-PAIC/TAT/PIC Kits (Sysmex Corporation Co., Ltd., Kobe, Japan), HISCL-800 Automated Chemiluminescence Immunoassay Analyzer; D-dimer/FIB Kits (Diagnostica Stago S.A.S. Co., Ltd., Asnières-sur-Seine, France), STA Compact Max Coagulation Analyzer |
| t-PAIC | ng/mL | 0-17 ng/mL | 1-100 ng/mL |  |  |
| TAT | ng/mL | 0-4 ng/mL | 0.4-120 ng/mL |  |  |
| PIC | μg/mL | 0-0.8 μg/mL | 0.05-40 μg/mL |  |  |
| D-dimer | μg/mL | 0-0.55 μg/mL | 0.19-4.8 μg/mL |  |  |
| FIB | g/L | 1.8-3.5 g/L | 0.8-7.0 g/L |  |  |
| Humoral immune parameters | | | | | |
| C3 | g/L | 0.9-1.8 g/L | 0.05-3.3 g/L | Immunoturbidimetric assays | C3/C4/Ig Kit (Meikang Biotechnology Co., Ltd., Ningbo, China); Siemens ADVIA 2400 Biochemical Analyzer |
| C4 | g/L | 0.16-0.38 g/L | 0.05-0.8 g/L |  |  |
| Serum IgA | g/L | 0.38-2.22 g/L | 0.5-35 g/L |  |  |
| Serum IgG | g/L | 5.9-14.3 g/L | 0.3-5 g/L |  |  |
| Serum IgM | g/L | 0.48-2.08 g/L | 0.04-5.5 g/L |  |  |
| Faecal occult blood test | | | | | |
| FOB | - | negative | 100-1000 ng/mL | Colloidal gold method | FOB Kits (Wuhan Kangzhu Biotechnology Co., Ltd., Wu Han, China), AVE Automated Stool Analyzer |

**Supplementary Table S2 Number of available observations for each laboratory parameter according to GI bleeding status**

| **Parameters** | **No GI bleeding group (n=82)** | **GI bleeding group (n=44)** | **Occult GI bleeding group (n=24)** | **Overt GI bleeding group (n=20)** |
| --- | --- | --- | --- | --- |
| WBC | 82 | 44 | 24 | 20 |
| ANC | 82 | 43 | 23 | 20 |
| ALC | 80 | 43 | 24 | 19 |
| NLR | 80 | 42 | 23 | 19 |
| sTM | 82 | 44 | 24 | 20 |
| t-PAIC | 82 | 44 | 24 | 20 |
| TAT | 82 | 44 | 24 | 20 |
| PIC | 82 | 44 | 24 | 20 |
| D-dimer | 82 | 44 | 24 | 20 |
| FIB | 82 | 43 | 23 | 20 |
| C3 | 81 | 43 | 23 | 20 |
| C4 | 81 | 43 | 23 | 20 |
| Serum IgA | 81 | 43 | 23 | 20 |
| Serum IgG | 81 | 43 | 23 | 20 |
| Serum IgM | 81 | 43 | 23 | 20 |

**Supplementary Table S3** Sensitivity analyses under alternative assumptions for children with undetermined GI-bleeding status

| **Analysis** | **Outcome definition** | **Analytic cohort** | **Variables** | **OR** | **95%CI** | ***P* value** |
| --- | --- | --- | --- | --- | --- | --- |
| Primary analysis | No-GI-bleeding group vs GI-Bleeding group; Undetermined GI-bleeding cases were excluded | n=126 | ANC | 1.097 | 0.970-1.241 | 0.140 |
|  |  |  | TAT | 0.956 | 0.903-1.013 | 0.130 |
|  |  |  | PIC | 1.888 | 1.013-3.518 | 0.045 |
|  |  |  | D-dimer | 1.191 | 0.998-1.422 | 0.053 |
|  |  |  | Serum IgG | 0.843 | 0.737-0.965 | 0.013 |
| Sensitivity analysis1 | All undetermined classified as no GI bleeding | n=162 | ANC | 1.120 | 1.008-1.244 | 0.036 |
|  |  |  | TAT | 0.969 | 0.923-1.017 | 0.198 |
|  |  |  | PIC | 1.649 | 0.982-2.768 | 0.059 |
|  |  |  | D-dimer | 1.052 | 0.919-1.205 | 0.462 |
|  |  |  | Serum IgG | 0.876 | 0.789-0.973 | 0.013 |
| Sensitivity analysis2 | All undetermined classified as GI bleeding | n=162 | ANC | 1.030 | 0.934-1.137 | 0.552 |
|  |  |  | TAT | 0.967 | 0.927-1.009 | 0.126 |
|  |  |  | PIC | 1.666 | 1.029-2.697 | 0.038 |
|  |  |  | D-dimer | 1.199 | 1.027-1.399 | 0.021 |
|  |  |  | Serum IgG | 0.913 | 0.831-1.003 | 0.058 |
